# Mathematical Modelling of Intravenous Thrombolysis in Acute Ischaemic stroke: Effects of Dose Regimens on Levels of Fibrinolytic Proteins and Clot Lysis Time

**DOI:** 10.3390/pharmaceutics11030111

**Published:** 2019-03-07

**Authors:** Boram Gu, Andris Piebalgs, Yu Huang, Colin Longstaff, Alun D. Hughes, Rongjun Chen, Simon A. Thom, Xiao Yun Xu

**Affiliations:** 1 Department of Chemical Engineering, Imperial College London, South Kensington Campus, London SW7 2AZ, UK; b.gu13@imperial.ac.uk (B.G.); andris.piebalgs@outlook.com (A.P.); y.huang@imperial.ac.uk (Y.H.); rongjun.chen@imperial.ac.uk (R.C.); 2 Biotherapeutics Section, National Institute for Biological Standards and Control, South Mimms, Hertfordshire EN6 3QG, UK; Colin.Longstaff@nibsc.org; 3 Institute of Cardiovascular Science, University College London, London WC1E 6DD, UK; alun.hughes@ucl.ac.uk; 4 MRC Unit for Lifelong Health and Ageing at University College London, London WC1B 5JU, UK; 5 National Heart & Lung Institute, Imperial College London, Hammersmith Campus, London W12 0NN, UK; s.thom@imperial.ac.uk

**Keywords:** thrombolysis, acute ischaemic stroke, tissue plasminogen activator, mathematical modelling, pharmacokinetics, pharmacodynamics

## Abstract

Thrombolytic therapy is one of the medical procedures in the treatment of acute ischaemic stroke (AIS), whereby the tissue plasminogen activator (tPA) is intravenously administered to dissolve the obstructive blood clot. The treatment of AIS by thrombolysis can sometimes be ineffective and it can cause serious complications, such as intracranial haemorrhage (ICH). In this study, we propose an efficient mathematical modelling approach that can be used to evaluate the therapeutic efficacy and safety of thrombolysis in various clinically relevant scenarios. Our model combines the pharmacokinetics and pharmacodynamics of tPA with local clot lysis dynamics. By varying the drug dose, bolus-infusion delay time, and bolus-infusion ratio, with the FDA approved dosing protocol serving as a reference, we have used the model to simulate 13 dose regimens. Simulation results are compared for temporal concentrations of fibrinolytic proteins in plasma and the time that is taken to achieve recanalisation. Our results show that high infusion rates can cause the rapid degradation of plasma fibrinogen, indicative of increased risk for ICH, but they do not necessarily lead to fast recanalisation. In addition, a bolus-infusion delay results in an immediate drop in plasma tPA concentration, which prolongs the time to achieve recanalisation. Therefore, an optimal administration regimen should be sought by keeping the tPA level sufficiently high throughout the treatment and maximising the lysis rate while also limiting the degradation of fibrinogen in systemic plasma. This can be achieved through model-based optimisation in the future.

## 1. Introduction

Blood clots can impair blood supply to major organs and cause life-threatening events, such as acute myocardial infarction (AMI), acute ischaemic stroke (AIS), and pulmonary embolism (PE). Thrombolysis is a medical treatment where a clot-busting drug is introduced to dissolve the obstructive clot, thereby restoring blood flow to ischaemic tissues. There are a number of thrombolytic agents available, e.g., streptokinase, alteplase, and urokinase. Alteplase is the first recombinant tissue plasminogen activator (rtPA or tPA) that was approved by the United States Food and Drug Administration (U.S. FDA) for the treatment of thromboembolic diseases [1], including AIS, following the National Institute of Neurological Disorders and Stroke (NINDS) trial [2]. Due to its high fibrin specificity, which is known to mitigate its systemic effects and haemorrhagic complications, it is currently the most commonly used agent [1]. The safety and efficacy of tPA drugs are dependent on many properties, of which clot specificity and half-life are most directly relevant to the effectiveness and risk of thrombolytic therapy. Furthermore, selecting an appropriate dose regimen is important for maximising the therapeutic efficacy whilst minimising the risk of serious complications. This requires insightful knowledge regarding the pharmacokinetics and pharmacodynamics (PKPD) of the drug during thrombolytic therapy.

A number of studies on the PKPD of tPA have been undertaken in searching for an optimal dosing regimen, both in healthy subjects and in patients predominantly with AMI [3,4,5,6,7,8,9,10]. Collen et al. conducted the first clinical trial in patients with four intravenous dosage regimens by using doses of tPA of 0.25 and 0.75 mg/kg and infusion durations of 15 and 120 min [6]. The results of thrombolysis and coagulant analysis confirmed that the use of tPA in coronary thrombolysis was beneficial without substantially depleting systemic fibrinogen levels, with 92 ± 4% of the original fibrinogen level remaining after tPA treatment. A later study using a single bolus of 50 mg in AMI patients showed that the simplified regimen achieved a comparable recanalisation rate to a standard regimen that comprises a 10-mg bolus, followed by 50 mg over one hour and 40 mg over two additional hours, although there was some evidence of incomplete thrombolysis and further assessment on haemorrhagic complications was needed [5].

Most of the clinical trials for thrombolysis in AMI were able to provide detailed PKPD data derived from the patients’ plasma samples, such as temporal plasma concentrations of tPA and fibrinolytic proteins, including fibrinogen, plasminogen, and α_2_-antiplasmin. These data can be used to elucidate the mechanism of fibrinolysis and the impact of dose regimen on therapeutic efficacy and toxicity. On the other hand, the clinical trials for AIS have focused on clinical and neurological outcomes, such as modified Rankin Scale and NIHSS [2,11,12,13,14,15,16]. Pathological differences between AMI and AIS may cause different responses to tPA dosage, making it difficult to directly apply the PKPD results on thrombolysis in AMI to AIS patients. Furthermore, AIS inherently exhibits a higher risk of intracranial haemorrhage (ICH) when compared to AMI due to acute brain infarction, which may be the reason for a lower dose of tPA recommended for the treatment of stroke than AMI [17]. Additionally, the approved dosage for treating AIS was determined based on clinical outcomes rather than PKPD studies [17]. Hence, there is a clear need for PKPD studies of tPA during thrombolytic therapy for AIS. However, clinical PKPD study on stroke patients would be impractical, as the treatment outcome is highly time-dependent [18]. Using a mathematical model can be very advantageous in such situations where access to experimental and clinical data is limited.

To the best of our knowledge, only two computational studies have attempted to include both the systemic effects of tPA via PK modelling and clot lysis dynamics [19,20]. Anand and Diamond [19], by combining a one-compartment model for the systemic circulation of tPA and a one-dimensional (1D) clot lysis model to simulate thrombolytic therapy for the treatment of AMI, developed a computational model. Their model provides a good foundation for a computational study of fibrinolysis and it accounts for crucial fibrinolytic proteins, although the description of fibrinolysis as a continuous shrinkage of fibre diameter seems unrealistic as fibrin fibres are cut transversely during fibrinolysis [21]. A more recent three-dimensional (3D) patient-specific model has been developed by Piebalgs et al. [20], which couples a one-compartment model and a fibrinolysis model that is based on the degradation of fibrin binding sites in the clot rather than fibre shrinkage. By using a multiscale modelling approach, the biochemical reactions of fibrinolysis are integrated with macroscale blood flow and drug transport in a 3D patient-specific model to assess the effects of different tPA doses on the outcome of thrombolytic therapy for AIS and the risk of ICH. Although the 3D model can capture more accurate haemodynamics and lysis patterns, it is computationally intensive, thus making it difficult to simulate a wide range of dosage regimens.

The present study aims to provide a mathematical modelling platform for computational simulation of thrombolytic therapy for the treatment of AIS. Our model includes (i) the systemic circulation and plasma reactions between fibrinolytic proteins (systemic PKPD) and (ii) clot lysis at the site of occlusion (local PD), whilst being computationally efficient for practical use. This model is developed upon the multiscale model of Piebalgs et al. [20], with the following modifications: (i) adding a second compartment to allow for the delayed clearance of tPA in the late phase of drug distribution and (ii) replacing the 3D blood flow and drug transport model with a reduced-order one-dimensional (1D) model for fast computation. The new model has been used to evaluate 13 different administration regimens for tPA and to compare recanalisation times with the reference case.

## 2. Methods

### 2.1. Overview of Modelling Strategy

**Figure 1:**
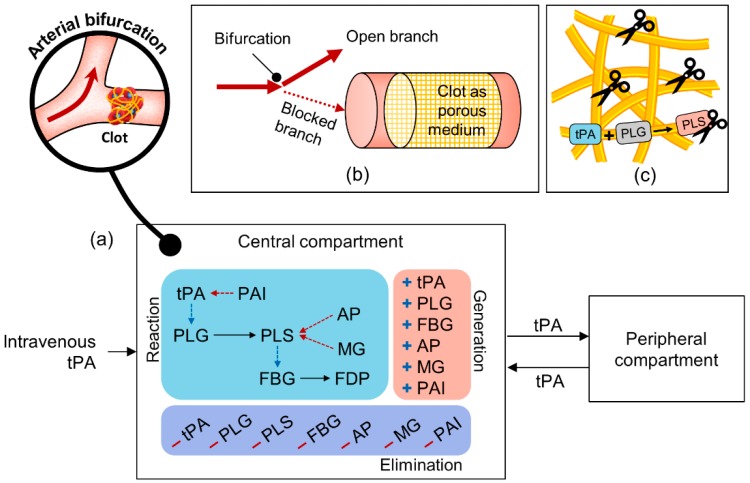
Overview of the full mathematical model. (**a**) Two-compartment model for pharmacokinetics and pharmacodynamics (PKPD) of tissue plasminogen activator (tPA) in the body (or the “systemic PKPD model”), (**b**) one-dimensional (1D) blood flow and species transport in an occluded artery, and (**c**) fibrinolysis described by binding of fibrinolytic proteins with fibrin fibres and the cleavage of fibrin network by bound plasmin. Coupled 1D blood flow and transport and fibrinolysis are referred to as the “local PD model”.

The full mathematical model comprises three submodels: (i) two-compartment model, (ii) 1D flow and species transport model, and (iii) fibrinolysis model, as shown in Figure 1a–c, respectively.

First, the two-compartment model is built to describe the systemic effects of tPA on fibrinolytic proteins in plasma following intravenous administration of tPA (PD), as well as the physiological processes, such as production and elimination of fibrinolytic proteins within the body (PK). As illustrated in Figure 1a, the body is described by two connected compartments. The central compartment represents the plasma where the distribution of tPA is almost instantaneous and six fibrinolytic proteins, plasminogen (PLG), plasmin (PLS), fibrinogen (FBG), α_2_-antiplasmin (AP), α_2_-macroglobulin (MG), and plasminogen activator inhibitor type 1 (PAI), are present in addition to tPA. The drug is administered to the central compartment and it moves between the central and peripheral compartments. It is assumed that movement of other fibrinolytic proteins between the two compartments is negligible, since the levels of these proteins in plasma are not expected to significantly change. Temporal concentrations of seven fibrinolytic proteins, including tPA, can be resolved using the compartmental model (or named as the “systemic PKPD model”) for a given dose regimen.

The drug and other fibrinolytic proteins are rapidly distributed within the central compartment, which also encompasses an arterial bifurcation that contains an occluded branch, e.g., the internal carotid bifurcation with the A1 segment of anterior cerebral artery and the occluded M1 segment of middle cerebral artery; one of the most common settings in stroke [22,23]. Since there is little blood flow in the occluded branch, the transport of fibrinolytic proteins is limited there. The amount of blood flow in the blocked artery can be determined by Darcy’s law based on clot permeability and pressure drop across the clot. Drug transport in the patent branch is assumed to be governed by the systemic PKPD model. Mass transport from the entrance of the blocked artery is described by the 1D convection-diffusion-reaction equations that contain source terms for reactions that take place both in the plasma and within the clot. In addition to the plasma reactions that are illustrated in Figure 1a, fibrinolytic reactions within the clot are incorporated, as depicted in Figure 1c; these include binding of tPA, PLG, and PLS onto the surfaces of the fibrin fibre network, activation of bound PLG into bound PLS, and fibrin degradation by bound PLS. We refer to the coupled 1D flow and transport model with the fibrinolysis kinetics model as the “local PD model”.

### 2.2. Mathematical Models

The model equations are outlined below, with a focus on critical modifications from the previous study [20].

#### 2.2.1. Systemic Pharmacokinetics and Pharmacodynamics (PKPD) Model

For the systemic PKPD model depicted in Figure 1a, a system of ordinary differential equations is built to resolve temporal concentrations of tPA and other fibrinolytic proteins (PLG, PLS, FBG, AP, MG, and PAI) in the central compartment, and tPA concentration in the peripheral compartment. As mentioned earlier, the peripheral distribution of tPA is significant due to a dramatic rise in plasma tPA level upon the intravenous (IV) administration of tPA. Temporal changes in the central and peripheral tPA levels are written as:

(1) $$\frac{dC_{c}}{dt}=\frac{I}{V_{c}M_{w,tPA}}-k_{el,tPA}C_{c}-k_{cp}C_{c}+k_{pc}C_{p}+S_{tPA}+R_{tPA}^{plasma}$$

(2) $$\frac{dC_{p}}{dt}=k_{cp}C_{c}-k_{pc}C_{p}$$

(3) $$C_{tPA,sys}=C_{c}$$

where *C* is the concentration, *I* is the infusion rate in mg/s, *V_c_* the volume of central compartment, *M_w,tPA_* is the molecular weight of tPA (= 59,042.3 g/mol for alteplase), *R* is the reaction term for generation or consumption via fibrinolytic reactions, *S* is the systemic secretion (by endothelial cells for tPA), *k_el_* is the elimination rate constant, and *k* is the distribution kinetics constants. Subscripts *sys*, *c*, and *p* denote the systemic, central, and peripheral compartments, respectively. The superscript *plasma* refers to reactions taking places in the plasma phase. For other fibrinolytic proteins, component balance equations can be written as:

(4) $$\frac{dC_{i,sys}}{dt}=-k_{el,i}C_{i,sys}+R_{i}^{plasma}+S_{i},\;\mathrm{at}\;i=\mathrm{PLG},\;\mathrm{PLS},\;\mathrm{AP},\;\mathrm{FBG},\;\mathrm{MG}\;\mathrm{and}\;\mathrm{PAI}$$

The elimination constants *k_el,i_* and secretion rates *S_i_* can be obtained while using half-life and the initial concentration of each component [20]. Reactions between the fibrinolytic proteins in the plasma are listed in Table 1. Details on the reaction kinetics equations and their parameters (plasma reactions 1 to 5 in Table 1) can be found in the Supporting Information (Table S1).

#### 2.2.2. Coupled Flow, Transport and Clot Lysis Model (“Local Pharmacodynamics Model”)

**Figure 2:**
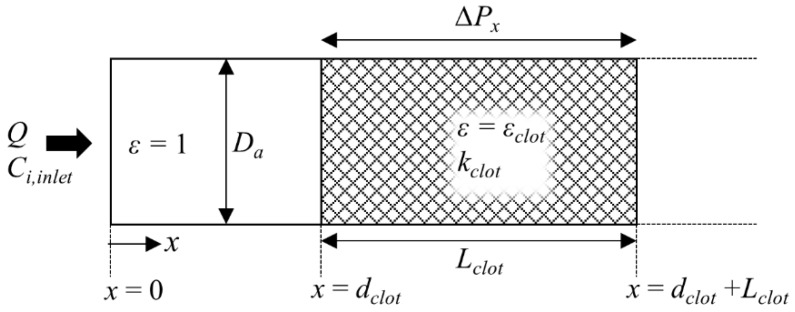
Schematic of 1D blood flow and species transport model. The entrance of the blocked artery corresponds to the bifurcating point where flow splits into two branches. The shaded area represents the clot with a porosity of *ε_clot_* and the open area is clot-free with *ε =* 1.

Along with key variables, 1D blood flow and species transport in an occluded artery are illustrated in Figure 2. Flow and species transport are unidirectional in the *x*-direction and so is the progression of clot dissolution. The artery is assumed to be circular with a diameter *D_a_*. The clot is treated as a porous medium with a length of *L_clot_*, a porosity of *ε_clot_*, and permeability of *k_clot_* and its front face is located at a distance of *d_clot_* away from the entrance of the occluded artery. Volumetric flowrate in the occluded artery *Q* is determined by the pressure drop per unit length across the clot ∆*P_x_*, which is dependent on the occlusion site and collateralisation [24].

According to Darcy’s law for flow through a porous medium, the volumetric flowrate *Q* can be obtained, as follows:

(5) $$Q=\frac{k_{clot}}{\mu }\Delta P_{x}$$

where *μ* is the fluid viscosity and the clot permeability *k_clot_* is calculated using Davies’ Equation [25]. The continuity equation for incompressible flow is employed.

(6) $$\frac{\partial Q}{\partial x}=0$$

Temporal and spatial variations of the concentration of protein *i* are expressed using the 1D convection-diffusion-reaction equations.

(7) $$\frac{\partial \varepsilon C_{i}}{\partial t}=-\frac{\partial \varepsilon UC_{i}}{\partial x}+D_{i}\frac{\partial^{2}\varepsilon C_{i}}{\partial x^{2}}+\varepsilon R_{i}^{tot}$$

(8) $$U=\frac{4Q}{\varepsilon \pi D_{a}^{2}}$$

where *U* is the flow velocity, *D_i_* the diffusivity of protein *i*, and *R_i_^tot^* is the sum of the rates of reactions taking place both in the plasma and clot that contribute to a change in *C_i_*, which can be obtained by:

(9) $$R_{i}^{tot}=R_{i}^{plasma}+R_{i}^{clot}$$

where *R^plasma^* and *R^clot^* are the rates of reactions in the plasma and in the clot, respectively, as listed in Table 1. The same model for clot lysis, as developed in the previous study [20], is employed here, and the reaction kinetics in the clot are summarised in Table 1. Temporal concentrations of bound phase proteins are also resolved using the reaction rates, as:

(10) $$\frac{\partial n_{j}}{\partial t}=R_{j}^{clot}\;\mathrm{for}\;j=\mathrm{tPA}\cdot F,\;\mathrm{PLG}\cdot F\;\mathrm{and}\;\mathrm{PLS}\cdot F$$

where *n_j_* is the concentration of bound phase protein *j*. Variation in the total concentration of binding sites *n_tot_* is determined by the degradation of binding sites by bound PLS [20].

(11) $$\frac{\partial n_{tot}}{\partial t}=-k_{deg}\gamma n_{PLS}$$

Based on the concentration of binding sites, the extent of lysis can be calculated, which is then used to estimate changes in porosity and permeability of clot [20]. Only the differential equations for the transport of free and bound phase proteins are repeated in this paper, and full model equations and parameter values can be found in the literature [20] or Supporting Information.

### 2.3. Model Integration and Numerical Procedure

**Figure 3:**
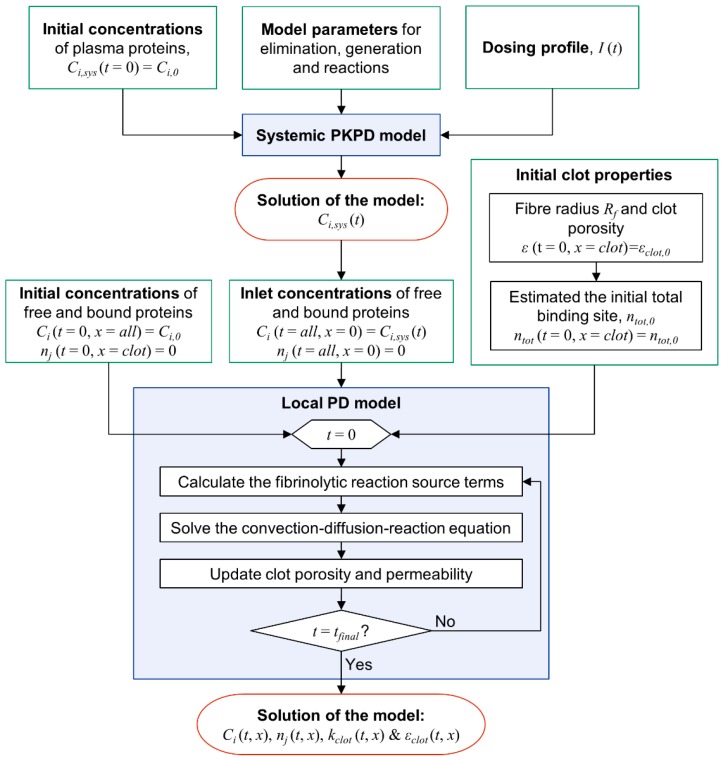
Flow chart of the solution procedure for the systemic and local models. Input information required for solving each model is specified in the green boxes.

The systemic PKPD model consists of a set of ordinary differential equations for temporal variations, while the local PD model is described by partial differential equations for both temporal and spatial variations. These equations are numerically solved through an in-house code that was programmed in MATLAB R2017a (The MathWorks, Inc., Natick, MA, United States). Figure 3 shows the flow chart of the numerical procedure employed. Initial and boundary conditions, as well as model parameters, are required as input.

The systemic PKPD model described by Equations (1–4) is solved at the prescribed initial concentrations of fibrinolytic proteins and drug dosing profile *I*(t) while using a MATLAB inbuilt solver for ordinary differential equations that employs the Runge–Kutta algorithm. In order to solve the local PD model, both initial and boundary conditions are required, as expressed in Equations (12) and (13).

(12) $$C_{i}\left(t,x\right)=C_{i,0}\;\mathrm{for}\;t=0\;\mathrm{and}\;x>0$$

(13) $$C_{i}\left(t,x\right)=C_{i,sys}\left(t\right)\;\mathrm{for}\;t>0\;\mathrm{and}\;x=0$$

Time-dependent concentrations of each fibrinolytic protein obtained from the systemic PKPD model, *C_i,sys_*, are then fed into the local PD model that is described by Equation (7) to serve as boundary conditions for the free phase proteins *C_i_*. Initial concentrations are assumed to be the same as those in the systemic PKPD model. The initial and boundary conditions for the bound phase proteins described by Equation (10) are set to zero, while the initial condition for the total concentration of binding sites in Equation (11) is:

(14) $$n_{tot}\left(t,x\right)=\{\begin{array}{ll} n_{tot,0}, & \mathrm{for}\;d_{clot}\leq x\leq d_{clot}+L_{clot}\\ 0, & \mathrm{otherwise} \end{array}\;\mathrm{at}\;t=0$$

where the initial total binding sites *n_tot,0_* is estimated using the average radius of the fibrin fibre [20]. The partial differential equations for all transporting species (Equation (7)) are numerically solved while using the finite difference method (a combination of the second order central, backward, and forward schemes) for spatial discretisation and backward Euler method for time integration. Detailed discretisation and integration procedures are included in Supporting Information, along with the results of grid independence study.

### 2.4. Simulation Details

Table 2 summarises the dosage regimens simulated here, including the FDA approved dosage regimen for alteplase as a reference case. Regimens 1 to 3 are designed to simulate different dose levels, while Regimens 4 to 6 cover the different delay intervals between bolus and continuous infusion. The amount of initial bolus, as introduced to boost the initial plasma drug level, is varied from 0% to 50% of the total dose (Regimens 7 to 9). Regimens 10 to 13 are chosen based on the protocols that were used for the treatment of AMI, i.e., a total dose of 100 mg with 15 mg, given as an initial bolus, 50 mg as continuous infusion for the first 30 min and 35 mg over the next 60 min.

The input parameters that were required to obtain model solutions are listed in Table 3. We assume that the clot is located in the M1 segment of the middle cerebral artery (MCA), which is a typical setting that is found in ischaemic stroke [22,23,26]. Initial concentrations of fibrinolytic proteins could be patient-dependent, which might alter the predicted treatment outcome and risk of ICH, especially due to substantial individual variations in the level of FBG [10]. Initial porosity and fibrin fibre radius are chosen to represent a platelet-retracted clot with a relatively coarse fibre network [24]. The total concentration of binding sites is estimated based on the fibre radius using the method that was developed in our previous work [20]. Pressure drop is chosen to be 60 mmHg/cm of clot length, a reported value for arterial occlusion, but it can vary depending on the degree of collateralisation [24]. The length and location of clot are based on clinical observations for MCA occlusions [22].

### 2.5. Remarks on Kinetics Parameters and Model Validation

Most of the kinetic parameters that were used in this study were derived from in vitro experimental data reported in the literature, as detailed in Table S1 (Supporting Information). The only exception was the kinetic parameter that is involved in the degradation of FBG, *k_3,cat_* in Table S1, which was adjusted to fit experimental findings. Prior to performing simulations for the selected dosage regimens, our model predictions were compared with several sets of PK data available in the literature [3,4,5,8]. Detailed comparisons can be found in Supporting Information (Section D).

## 3. Results and Discussion

### 3.1. Effects of tPA Dose on Systemic Concentrations of Thrombolytic Proteins

**Figure 4:**
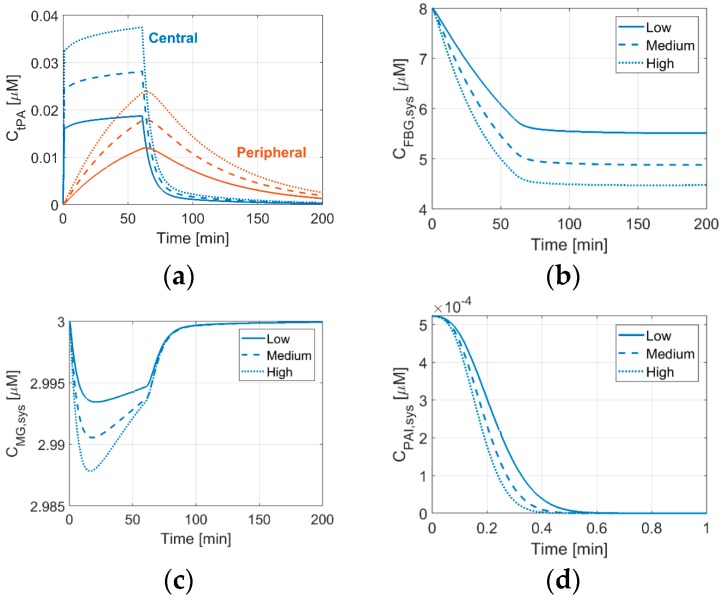
Changes in systemic concentrations of fibrinolytic proteins over time with different drug doses: 0.6 (Regimen 2), 0.9 (Regimen 1), and 1.2 (Regimen 3) mg/kg of patient weight, as low (solid line), medium (dashed line) and high (dotted line) doses, respectively. (**a**) tPA concentration in the central (blue lines) and peripheral (orange lines) compartments, (**b**) systemic concentrations of fibrinogen (FBG), (**c**) α_2_-macroglobulin (MG), and (**d**) plasminogen activator inhibitor type 1 (PAI) (shown for 1 min only).

Figure 4 shows temporal concentrations of tPA in the central and peripheral compartments, as well as systemic concentrations of FBG, MG, and PAI that were obtained from the two-compartment model. The concentration profiles of tPA and FBG are of particular importance, as they are indicative of therapeutic efficacy and risk of ICH, respectively. MG and PAI are the new proteins that are included in the two-compartment model developed in this study.

As can be seen in Figure 4a, the tPA concentration in central plasma rises sharply upon the initial bolus administration, while its level in the peripheral compartment gradually picks up until the infusion stops and then falls slowly. Including the peripheral compartment in the systemic PKPD model results in a slight increase in plasma tPA during continuous infusion, which was not captured by the one-compartment model [20]. In addition, the plasma tPA level more slowly drops with the two-compartment model. However, the temporal profile of FBG concentration that is predicted by the current model is similar to that of the one-compartment model [20], showing a steady reduction in FBG during drug administration. With an initial FBG level of 8 μM, the final level of FBG lies between 4.5 and 5.5 μM, depending on the tPA dose; equivalent to around 30–45% reduction from its initial level. This means that a further increase of tPA dose might cause the FBG level to fall below 150 mg/dL (equivalent to around 4.4 μM), which is a reported threshold value for increased risk of bleeding [29]. Due to substantial individual variations in FBG concentration (5–13 μM) [10], some patients with a low initial FBG level may be vulnerable to ICH, even at a low tPA dose.

For the predicted MG concentration, its temporal variation is negligible, as seen in Figure 4c. This is because, when compared to AP, which is the main inhibitor of PLS [3,30], MG is a relatively weak inhibitor. PAI also has a negligible role due to its low concentration in plasma. PAI is depleted very quickly, within less than 1 min, as shown in Figure 4d, and its concentration rises very slowly after the treatment is finished (not shown in the figure).

### 3.2. Effects of Delays between Bolus and Continuous IV Infusion on Systemic Concentrations

**Figure 5:**
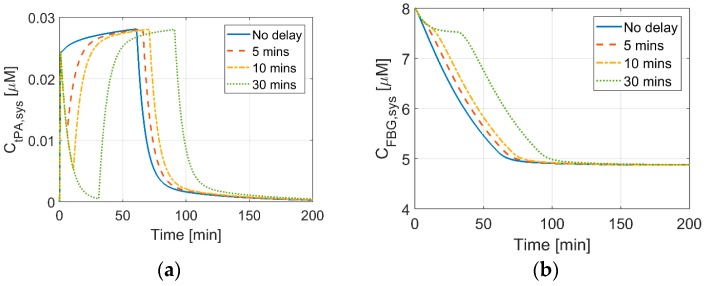
Effects of bolus-continuous IV infusion delay on the concentrations of (**a**) tPA and (**b**) FBG. Simulated Regimens 4–6 correspond to delays of 5, 10, and 30 min, respectively. The reference regimen with no delay (Regimen 1) is also included for comparison.

Effects of delay intervals between bolus and continuous infusion are shown in Figure 5, where results for delays of 5 min (Regimen 4), 10 min (Regimen 5), and 30 min (Regimen 6) are compared with the reference case with no delay. It shows clearly that, in the absence of a time delay, the plasma tPA concentration stays at a relatively constant level throughout the treatment. When a delay is introduced before continuous infusion, the tPA level rapidly drops after the bolus injection due to its short half-life (around 4 to 5 min) and it starts to increase again once the continuous infusion is administered. Increasing the delay between the bolus and continuous infusion reduces the minimum level of tPA and it prolongs the time period when the tPA concentration is below its therapeutic level. These results are in accordance with those reported by Smith et al. [31], who recommended an additional bolus injection to restore plasma tPA level in the case of any bolus-infusion delay.

Figure 5b shows that the length of bolus-infusion delay does not affect the final level of FBG at the end of the treatment, so long as the total dose is kept the same. This suggests that the rate of degradation and final level of FBG are independent of dosing schedule. As expected, increasing the bolus-infusion delay would delay the start of FBG degradation.

### 3.3. Effects of Bolus to Continuous Infusion Ratio on Systemic Concentrations

**Figure 6:**
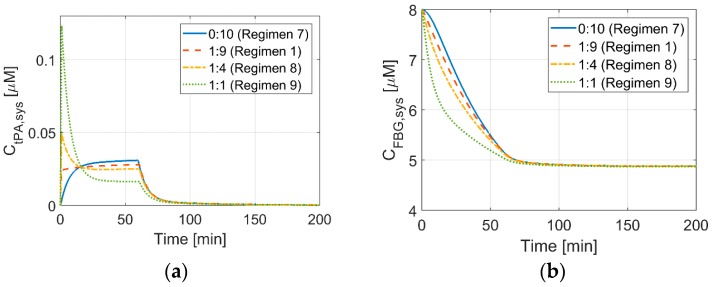
Effects of bolus to infusion ratios at a fixed dose of 0.9 mg/kg, on the concentration of (**a**) tPA, and (**b**) FBG. The amount of bolus is varied from 0% (Regimen 7) to 50% (Regimen 9) of the total dose. Regimen 1 has a bolus to infusion ratio of 1:9, while Regimens 8 and 9 have a ratio of 1:4 and 1:1, respectively.

Figure 6 shows simulation results for different bolus-infusion ratios (Regimens 1 and 7–9) at a fixed total dose. As the amount of bolus increases, the initial tPA concentration rises, reaching 0.12 μM when 50% of the total dose is administered as bolus, which is about five times higher than the initial tPA concentration that was achieved by the standard regimen. When the full dose is administered via continuous IV infusion without an initial bolus injection (Regimen 7), the level of tPA in plasma gradually increases and it reaches 0.025 μM at around 15 min, which is approximately the steady state tPA level corresponding to the standard dosage regimen (Regimen 1).

Due to the high tPA concentration at high bolus ratios, FBG is depleted rapidly in the initial stage, as shown in Figure 6b. This suggests that there might be an increased risk of ICH at the early stage of treatment when a large amount of bolus is administered to patients with a low FBG level. Nonetheless, the final level of FBG at the termination of drug infusion is the same for all of the simulated scenarios.

Tebbe et al. studied a single bolus injection of 50 mg of tPA for the treatment of AMI with an aim to simplify the therapeutic regimen that usually involves several steps [5]. They also reported approximately a three-fold increase in peak tPA concentration relative to the conventional regimen. However, they observed high rates of recurring ischaemic events, possibly due to the lower total dose administered (50 mg) when compared to the conventional regimen (100 mg) for AMI.

### 3.4. Systemic Concentrations of Thrombolytic Proteins for New Dosage Regimens

**Figure 7:**
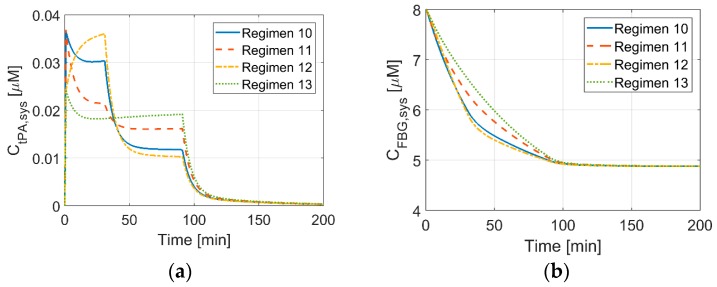
Concentrations of tPA (**a**) and FBG (**b**)over time for the new regimens simulated in this study.

New dosage regimens, which are based on the administration regimen for accelerated treatment of AMI, are simulated. The results for tPA and FBG concentrations are shown in Figure 7a,b, respectively. For the same amount of bolus, the initial tPA level is the same, i.e., 0.037 μM for Regimens 10 and 11 and 0.024 μM for Regimens 12 and 13. When the bolus injection is stopped, the tPA level decreases for all of the Regimens, except for Regimen 12, where 60% of the total dose is continuously infused over 30 min straight after the initial bolus. For Regimens 11 and 13, where the largest portion of the total dose is infused over the final hour of the treatment, changes in tPA concentration are less dramatic when compared to the other regimens. The initial and final FBG levels are the same for all four regimens, but there is a slight difference in the reduction rate. When plasma tPA level is relatively steady throughout the treatment, the FBG level tends to drop more gradually, e.g., Regimen 13. This suggests that dosage regimens that can maintain a relatively constant plasma tPA level may help to lower the risk of ICH by reducing the duration when patients are exposed to low FBG levels.

### 3.5. Effects of Different Dosage Regimens on Recanalisation Time

**Figure 8:**
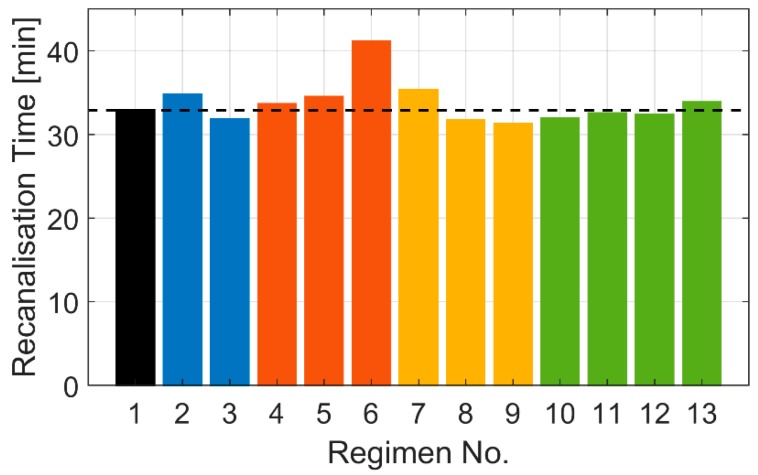
Predicted recanalisation times for the simulated dosage regimens. The black bar and dashed line are the reference regimen. Different colour bars indicate the therapeutic parameters that are varied: tPA dose (blue bars), bolus-infusion delay (orange), bolus-infusion ratio (yellow), and new regimens (green).

Figure 8 summarises the predicted recanalisation times for different dosage regimens. It shows that recanalisation is achieved within 31 to 41 min for all simulated regimens. These results are consistent with reported reperfusion times of between 20 to 80 min for AMI [25] and 23 ± 16 min for AIS [32].

The fastest clot lysis is achieved by Regimen 9, which has the highest bolus percentage. The large amount of initial bolus allows for a high level of tPA to be maintained for up to 25 min, as shown in Figure 6a, during which clot lysis is almost accomplished for the simulated clot. Nevertheless, a large amount of initial bolus can increase the risk of ICH from the start of the treatment, as shown in Figure 6b, especially for patients with low plasma FBG level. Hence, there is a trade-off between fast recanalisation and elevated risk of ICH.

The effects of the bolus-continuous infusion time delays on recanalisation time are more pronounced (Regimens 4 to 6), with up to 8 min difference in achieving complete clot lysis between no delay and 30 min bolus-infusion delay. Since the clot in the simulated scenario is relatively easy to dissolve with the standard dosage regimen without having to use the whole prescribed dose, the effect of bolus-infusion delay on recanalisation time is expected to be less marked for denser clots that are harder to dissolve. This explanation also applies to the results for Regimens 2 and 3, with different tPA doses. Even with a low dose (Regimen 2), the complete clot lysis only takes 2 min longer than with the standard dose. Moreover, our results show that increasing the tPA dose appears to achieve a relatively small gain in recanalisation time for the simulated clot. This can be explained by the dramatic reduction in PLG concentration at a high tPA dose after continuous infusion [20], i.e., the final PLG level at the high dose (1.2 mg/kg) is half of that at the low dose (0.6 mg/kg). Therefore, it is believed that high tPA doses do not always directly correlate to accelerated lysis and an optimal drug dose should be determined based on clot properties, such as its length, location, composition, and permeability. This has also been corroborated by several clinical studies, where the use of a low dose at 0.6 mg/kg achieved successful recanalisation, a comparable treatment outcome, and similar or reduced incidence of ICH [13,33,34].

### 3.6. Temporal and Spatial Variations in Protein Concentration

**Figure 9:**
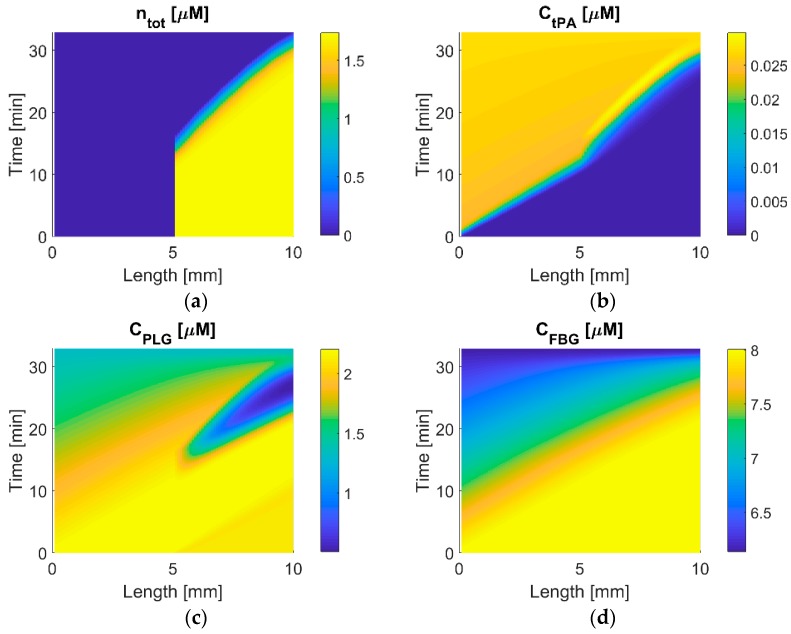
Temporal and spatial concentrations of free phase tPA, PLG, and FBG, and the total concentration of binding sites (*n_tot_*) in the clot for the standard dosage regimen. Each map shows variations of concentration in time (vertical axis) and space (horizontal axis). (**a**) The total binding site and (**b**) free phase tPA, (**c**) PLG, and (**d**) FBG.

Distributions of free phase tPA, PLG, and FBG, and the total binding sites are shown in Figure 9. The clot is located at *x* = 5 to 10 mm, which can be identified from the total binding sites map, i.e., *n_tot_* > 0. During the initial phase (up to around 12 min), there is a homogenous distribution of the total binding sites within the clot, as shown in Figure 9a. Free tPA in plasma is transported at a constant rate before reaching the clot, as shown in Figure 9b. When the amount of drug at the clot front reaches the therapeutic level, the clot starts to degrade (at around 12 min), until the complete dissolution (at around 30 min). During the period of clot lysis, tPA slowly penetrates into the clot, creating a band of high tPA concentration near the clot front. Diamond and Anand have also obtained similar tPA spatial profiles with high concentration gradient at the lysis front [25].

The PLG concentration map exhibits an interesting pattern, as shown in Figure 9c; there is a valley of low PLG concentration near the lysis front, where free PLG is rapidly consumed by the fibrinolytic reactions taking place in plasma as well as binding with the fibrin network. This leads to a slight deceleration at the late stage of clot lysis, as can be seen in *n_tot_* around 30 min in Figure 9a. For the FBG concentration that is shown in Figure 9d, there is no distinct pattern apart from the reduction in FBG concentration from 8 to around 6.1 μM over time due to the systemic action of tPA on FBG.

## 4. Conclusions and Future Perspectives

We have developed a computationally efficient simulation platform for thrombolysis in ischaemic stroke in order to evaluate the therapeutic efficacy under different treatment protocols. Our mathematical model accounts for the systemic effects of thrombolytic drug and 1D progression of clot lysis, so that the lysis completion time and plasma FBG level (a measure to assess the risk of ICH) can be predicted without excessive computational demands. The model is capable of simulating various therapeutic scenarios by choosing a relevant set of PK and kinetics parameters, and it can be readily extended to simulate different fibrinolytic drugs other than alteplase. It can also be applied to a variety of AIS scenarios by changing the geometric parameters in the local PD model and clot properties that are based on patient-specific information. In the future, the model will be extended to examine the effects of various therapeutic and physiological factors on recanalisation rate, and to design personalised dosing regimen via model-based optimisation.

## Figures and Tables

**Table 1:** List of reactions between fibrinolytic proteins in the plasma and clot.

	No.	Description
Plasma	1	$\mathrm{tPA}+\mathrm{PLG}\overset{K_{1,M}\;\&\;k_{1,cat}}{\to }\mathrm{tPA}+\mathrm{PLS}$
	2	$\mathrm{PLS}+\mathrm{AP}\overset{k_{2,f}}{\underset{k_{2,r}}{⇄}}\mathrm{PLS}\cdot \mathrm{AP}\overset{k_{2,cat}}{\to }\mathrm{inactive}$
	3	$\mathrm{PLS}+\mathrm{FBG}\overset{K_{3,M}\;\&\;k_{3,cat}}{\to }\mathrm{PLS}+\mathrm{FDP}$
	4	$\mathrm{PLS}+\mathrm{MG}\overset{k_{4}}{\to }\mathrm{inactive}$
	5	$\mathrm{tPA}+\mathrm{PAI}\overset{k_{5}}{\to }\mathrm{inactive}$
Clot	1	$\mathrm{tPA}+F\overset{k_{a,tPA}}{\underset{k_{d,tPA}}{⇄}}\mathrm{tPA}\cdot F$
	2	$\mathrm{PLG}+F\overset{k_{a,PLG}}{\underset{k_{d,PLG}}{⇄}}\mathrm{PLG}\cdot F$
	3	$\mathrm{PLS}+F\overset{k_{a,PLS}}{\underset{k_{d,PLS}}{⇄}}\mathrm{PLS}\cdot F$
	4	$\mathrm{tPA}\cdot F+\mathrm{PLG}\cdot F\overset{K_{M}\;\&\;k_{M,cat}}{\to }\mathrm{tPA}\cdot F+\mathrm{PLS}\cdot F$
	5	$\mathrm{PLS}\cdot F\overset{k_{deg}}{\to }\mathrm{PLS}\cdot \tilde{F}$
	6	$\mathrm{PLS}\cdot \tilde{F}\overset{k_{d,PLS}}{\to }\mathrm{PLS}+\tilde{F}$

**Table 2:** List of simulated dosage regimens. Regimen 1 is the standard dose for the treatment of ischaemic stroke.

No.	Total Dose	Regimen Description
1	0.9 mg/kg	10% as bolus + 90% as continuous over 1 h (reference case)
2	0.6 mg/kg	10% as bolus + 90% as continuous over 1 h
3	1.2 mg/kg	10% as bolus + 90% as continuous over 1 h
4	0.9 mg/kg	10% as bolus + 5-min delay + 90% as continuous over 1 h
5	0.9 mg/kg	10% as bolus + 10-min delay + 90% as continuous over 1 h
6	0.9 mg/kg	10% as bolus + 30-min delay + 90% as continuous over 1 h
7	0.9 mg/kg	the total dose as a continuous infusion over 1 h
8	0.9 mg/kg	20% as bolus + 80% as continuous over 1h
9	0.9 mg/kg	50% as bolus + 50% as continuous over 1h
10	0.9 mg/kg	15% as bolus + 50% as continuous over 30 min + 35% as continuous over 1 h
11	0.9 mg/kg	15% as bolus + 35% as continuous over 30 min + 50% as continuous over 1 h
12	0.9 mg/kg	10% as bolus + 60% as continuous over 30 min + 30% as continuous over 1 h
13	0.9 mg/kg	10% as bolus + 30% as continuous over 30 min + 60% as continuous over 1 h

**Table 3:** List of input parameters and their values that were used in this work.

Symbol	Definition	Values [unit]	Source
*C_tPA,0_*	Initial tPA concentration	0.05 [nM]	[24]
*C_PLG,0_*	Initial PLG concentration	2.2 [μM]	[24]
*C_PLS,0_*	Initial PLS concentration	0 [μM]	[24]
*C_FBG,0_*	Initial FBG concentration	8 [μM]	[24]
*C_AP,0_*	Initial AP concentration	1 [μM]	[24]
*C_MG,0_*	Initial MG concentration	3 [μM]	[24]
*C_PAI,0_*	Initial PAI concentration	5.23 × 10^−4^ [μM]	[27]
*ε_clot,0_*	Initial porosity of the clot	0.95 [-]	[24]
*R_f_*	Radius of fibrin fibre in the clot	100 [nm]	[24]
*μ*	Blood viscosity	0.0037 [Pa∙s]	[20]
*n_tot,0_*	Initial total concentration of binding sites in the clot	1.74 [μM]	[20]
∆*P_x_*	Pressure drop per unit length across the clot	60 [mmHg/cm]	[24]
*d_clot_*	Distance from the bifurcation to the clot front	5 [mm]	[26]
*L_clot_*	Length of clot	5 [mm]	[22]
*D_a_*	Diameter of occluded artery	3 [mm]	[28]

## Supplementary Materials

The following are available online at https://www.mdpi.com/1999-4923/11/3/111/s1, Figure S1: Illustration of space and time discretisation, Figure S2: Data presented in Tienfenbrunn et al. with 25 mg of tPA infused over 7 h, Figure S3: Data presented in Tienfenbrunn et al. with 60 mg of tPA infused over 90 min, Figure S4: Data presented in Collen et al. with 0.75 mg/kg of tPA over 90 min, Figure S5: Data presented in Tanswell et al. with tPA dose of 0.25 mg/kg over 30 min, Figure S6: Data presented in Tanswell et al. with tPA dose of 0.5 mg/kg over 30 min, Figure S7: Data presented in Tebbe et al with a single bolus of 50 mg of tPA over 2 min, Table S1: Summary of reactions between plasma proteins in the systemic PKPD model, Table S2: Additional parameter values for the new compartment model, Table S3: Summary of grid test results.
