# Supplementary material for: Mathematical Modelling of Intravenous Thrombolysis in Acute Ischaemic stroke: Effects of Dose Regimens on Levels of Fibrinolytic Proteins and Clot Lysis Time
Source: Pharmaceutics. 2019 Mar 7;11(3):111. doi: 10.3390/pharmaceutics11030111 (PMC6471481; doi:10.3390/pharmaceutics11030111)
Supplement: Supplementary file 1 [file pharmaceutics-11-00111-s001.pdf]

pharmaceutics

MDPI

Supplementary Materials: Mathematical Modelling of Intravenous Thrombolysis in Acute Ischaemic stroke: Effects of Dose Regimens on Levels of Fibrinolytic Proteins and Clot Lysis Time

Boram Gu, Andris Piebalgs, Yu Huang, Colin Longstaff, Alun D. Hughes, Rongjun Chen, Simon A. Thom and Xiao Yun Xu

A. Model Equations and Parameters

A.1. Model Equations for Reaction Kinetics

Based on the reaction kinetics presented in Table 1, reaction source terms for each species in the systemic reactions and clot lysis can be written as follows.

$$R_{tPA}^{plasma} = -k_5 C_{tPA} C_{PAI} \quad \text{(A1)}$$

$$R_{PLG}^{plasma} = -\frac{k_{1,cat} C_{tPA} C_{PLG}}{K_{1,M} + C_{PLG}} \quad \text{(A2)}$$

$$R_{PLS}^{plasma} = \frac{k_{1,cat} C_{tPA} C_{PLG}}{K_{1,M} + C_{PLG}} - k_{2,f} C_{PLS} C_{AP} + k_{2,r} C_{PLS?AP} - k\ C_{PLS} C_{MG} \quad \text{(A3)}$$

$$R_{FBG}^{plasma} = -\frac{k_{3,cat} C_{PLS} C_{FBG}}{K_{3,M} + C_{FBG}} \quad \text{(A4)}$$

$$R_{AP}^{plasma} = -k_{2,f} C_{PLS} C_{AP} + k_{2,r} C_{PLS?AP} \quad \text{(A5)}$$

$$R_{PLS?AP}^{plasma} = k_{\ ,f} C_{PLS} C_{AP} - k_{2,cat} C_{PLS?AP} \quad \text{(A6)}$$

$$R_{MG}^{plasma} = -k_4 C_{PLS} C_{MG} \quad \text{(A7)}$$

$$R_{PAI}^{plasma} = -k_5 C_{tPA} C_{PAI} \quad \text{(A8)}$$

$$R_{tPA}^{clot} = -k_{a,tPA} C_{tPA} n_{free} + k_{d,tPA} n_{tPA} \quad \text{(A9)}$$

$$R_{tPA\cdot F}^{clot} = k_{a,tPA} C_{tPA} n_{free} - k_{d,tPA} n_{tPA} \quad \text{(A10)}$$

$$R_{PLG}^{clot} = -k_{a,PLG} C_{PLG} n_{free} + k_{d,PLG} n_{PLG} \quad \text{(A11)}$$

$$R_{PLG?F}^{clot} = k_{a\ PLG} C_{PLG} n_{free} - k_{d,PLG} n_{PLG} + \frac{k_{M,cat} n_{tPA} n_{PLG}}{K_M \left(1 - \varepsilon_{clot}\right) + n_{PLG}} \quad \text{(A12)}$$

$$R_{PLS}^{clot} = -k_{a,PLS} C_{PLS} n_{free} + k_{d,PLS} n_{PLS} + k_{r,PLS} n_{PLS\cdot\tilde{F}} \quad \text{(A13)}$$

$$R_{PLS?F}^{clot} = k_{a\ PLS} C_{PLS} n_{free} - k_{d,PLS} n_{PLS} + \frac{k_{M,cat} n_{tPA} n_{PLG}}{K_M \left(1-\varepsilon_{clot}\right) + n_{PLG}} - k_{deg} \gamma n_{PLS} \quad \text{(A14)}$$

$$R_{PLS\cdot\tilde{F}}^{clot} = k_{deg} \gamma n_{PLS} - k_{d,PLS} n_{PLS\cdot\tilde{F}} \quad \text{(A15)}$$

The reaction source terms for the plasma proteins in Equations (A1–A8) are used in Equations (1) and (4) in the systemic PKPD model. For the local PD model, all the above equations are required to calculate the total reaction rates R_i^{tot} in Equation (9), which is then fed to Equations (7) and (10) for temporal and spatial concentrations of free and bound phase proteins. The concentration of free binding sites is calculated as:

$$n_{free} = n_{tot} - n_{tPA} - n_{PLG} - n_{PLS} \quad \text{(A16)}$$

and n_{tot} is obtained by solving Equation (11).

Kinetics parameters for the systemic PKPD model are listed in Table S1 with their sources, while kinetic parameters for the reactions within the clot can be found in our previous work [S1]. Reactions 1 and 3 are described by the Michaelis-Mention kinetics, and their parameters $K_{3,M}$ and $k_{3,cat}$ in Table 1 are derived under the assumption of quasi-steady state using the forward and reverse constants and catalytic reaction constant in Table S1.

Table S1. Summary of reactions between plasma proteins in the systemic PKPD model.

No.	Reaction	Parameter [unit]	Source
1	$\text{tPA} + \text{PLG} \underset{k_{1,r}}{\overset{k_{1,f}}{\rightleftarrows}} \text{tPA}\cdot\text{PLG} \xrightarrow{k_{1,cat}} \text{tPA} + \text{PLS}$	$k_{1,f}$ = 10 [1/(μM·s)]<br>$k_{1,r}$ = 280 [1/s]<br>$k_{1,cat}$ = 0.3 [1/s]	[S2]
2	$\text{PLS} + \text{AP} \underset{k_{2,r}}{\overset{k_{2,f}}{\rightleftarrows}} \text{PLS}\cdot\text{AP} \xrightarrow{k_{2,cat}} \text{inactive}$	$k_{2,f}$ = 10 [1/(μM·s)]<br>$k_{2,r}$ = 0.0021 [1/s]<br>$k_{2,cat}$ = 0.004 [1/s]	[S2]
3	$\text{PLS} + \text{FBG} \underset{k_{3,r}}{\overset{k_{3,f}}{\rightleftarrows}} \text{PLS}\cdot\text{FBG} \xrightarrow{k_{3,cat}} \text{PLS} + \text{FDP}$	$k_{3,f}$ = 10 [1/(μM·s)]<br>$k_{3,r}$ = 300 [1/s]<br>$k_{3,cat}$ = 25 [1/s] or 250 [1/s]	[S2]
4	$\text{PLS} + \text{MG} \xrightarrow{k_4} \text{inactive}$	k_4 = 0.35 [1/(μM·s)]	[S3]
5	$\text{tPA} + \text{PAI} \xrightarrow{k_5} \text{inactive}$	k_5 = 37 [1/(μM·s)]	[S4]

A.2. Additional Equations used in the Model

Elimination constant, $k_{el,i} = \dfrac{\ln 2}{t_{1/2,i}}$ (A17)

Generation rate, $S_i = k_{el,i} C_{i,0}$ (A18)

Extent of lysis, $E_L = 1 - \dfrac{n_{tot}}{n_{tot,0}}$ (A19)

Volume fraction of fibrin fibre network in the clot, $\phi_f = \phi_{f,0}\left(1 - E_L\right)$ (A20)

Porosity of clot, $\varepsilon_{clot} = 1 - \phi_f$ (A21)

Permeability of clot, $$k_{clot} = \begin{cases} \dfrac{4R_f^{\,2}}{70\left(1-\varepsilon_{clot}\right)^{1.5}\left[1+52\left(1-\varepsilon_{clot}\right)^{1.5}\right]} & \text{when } E_L < E_{L,crit} \\ \infty & \text{when } E_L \geq E_{L,crit} \end{cases} \quad \text{(A22)}$$

Parameters that are introduced in the new model are listed in Table S2 and those that are not here can be found in the previous work [1].

Table S2. Additional parameter values for the new compartment model.

Symbol	Value	Units	Source
$t_{1/2,PAI}$	1.5	h	[S5]
$t_{1/2,MG}$	3	min	[S6]
$k_{el,tPA}$	2.27×10^{-3}	1/s	[S7]
k_{12}	3.10×10^{-4}	1/s	[S7]
k_{21}	3.34×10^{-4}	1/s	[S7]
V_c	0.057	L/kg of patient weight	[S7]

B. Numerical Procedures for 1D Transport and Clot Lysis Model ("Local PD Model")

The local PD model, presented in Section 2.2.2, is solved using the finite difference scheme. The 1D domain of the artery where the clot is located is discretised into small cells with a uniform cell size of Δx, as shown in Figure S1. The 1st order space derivative is approximated by the 2nd order backward differencing scheme, except for the first cell (j = 1) where the 2nd order forward difference scheme is used. The 2nd order space derivative is approximated by the central differencing scheme. For time integration, the backward Euler method is used.

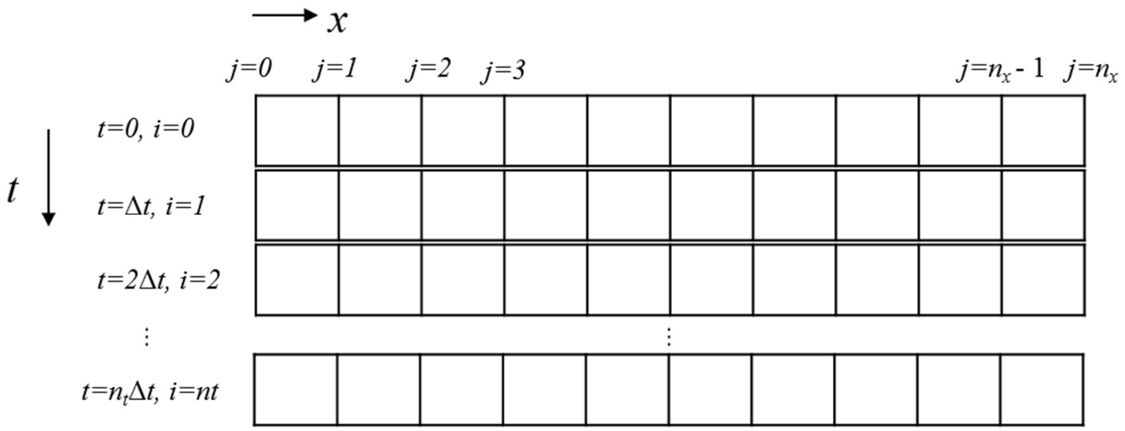

Figure S1. Illustration of space and time discretisation.

Therefore, Equation (7) can be expanded to a set of algebraic equations for time i, shown below:

$$\frac{C(i,1)-C(i-1,1)}{\Delta t}+U(i-1)\frac{-3C(i,0)+4C(i,1)-C(i,2)}{2\Delta x}-D\frac{C(i,0)-2C(i,1)+C(i,2)}{\Delta x^2}=R(i-1,1)$$

$$\frac{C(i,2)-C(i-1,2)}{\Delta t}+U(i-1)\frac{3C(i,2)-4C(i,1)+C(i,0)}{2\Delta x}-D\frac{C(i,1)-2C(i,2)+C(i,3)}{\Delta x^2}=R(i-1,2)$$

$$\vdots$$

$$\frac{C(i,n_x-1)-C(i-1,n_x-1)}{\Delta t}+U(i-1)\frac{3C(i,n_x-1)-4C(i,n_x-2)+C(i,n_x-3)}{2\Delta x}-D\frac{C(i,n_x-2)-2C(i,n_x-1)+C(i,n_x)}{\Delta x^2}=R(i-1,n_x-1)$$

(B1)

By applying a zero gradient boundary condition at the end of clot, a concentration term at the last point $C(i,n_x)$ can be expressed as:

$$C(1:n_t, n_x) = \frac{4C(1:n_t, n_x-1) - C(1:n_t, n_x-2)}{3} \tag{B2}$$

and the last equation for j=n_x–1 in Eq (B1) becomes:

$$\frac{C(i,n_x-1)-C(i-1,n_x-1)}{\Delta t} + U(i-1)\frac{3C(i,n_x-1)-4C(i,n_x-2)+C(i,n_x-3)}{2\Delta x} - D\frac{C(i,n_x-2)-2C(i,n_x-1)+\dfrac{4C(i,n_x-1)-C(i,n_x-2)}{3}}{\Delta x^2} = R(i-1,n_x-1) \tag{B3}$$

The above equations are arranged in a way that the right-hand side consists of information on the previous time steps.

$$\begin{aligned}
\left(\frac{1}{\Delta t}+\frac{4U}{2\Delta x}+\frac{2D}{\Delta x^2}\right)C(i,1)+\left(-\frac{U}{2\Delta x}-\frac{D}{\Delta x^2}\right)C(i,2) &= R(i-1,1)+\frac{C(i-1,1)}{\Delta t}+U\frac{3C(i,0)}{2\Delta x}+D\frac{C(i,0)}{\Delta x^2}\\
\left(-\frac{4U}{2\Delta x}-\frac{D}{\Delta x^2}\right)C(i,1)+\left(\frac{1}{\Delta t}+\frac{3U}{2\Delta x}+\frac{2D}{\Delta x^2}\right)C(i,2)+\left(-\frac{D}{\Delta x^2}\right)C(i,3) &= R(i-1,2)+\frac{C(i-1,2)}{\Delta t}-U\frac{C(i,0)}{2\Delta x}\\
\left(\frac{U}{2\Delta x}\right)C(i,1)+\left(-\frac{4U}{2\Delta x}-\frac{D}{\Delta x^2}\right)C(i,2)+\left(\frac{1}{\Delta t}+\frac{3U}{2\Delta x}+\frac{2D}{\Delta x^2}\right)C(i,3)+\left(-\frac{D}{\Delta x^2}\right)C(i,4) &= R(i-1,3)+\frac{C(i-1,3)}{\Delta t}\\
&\vdots\\
\left(\frac{U}{2\Delta x}\right)C(i,n_x-3)+\left(-\frac{4U}{2\Delta x}-\frac{D}{\Delta x^2}+\frac{D}{3\Delta x^2}\right)C(i,n_x-2)+\left(\frac{1}{\Delta t}+\frac{3U}{2\Delta x}+\frac{2D}{\Delta x^2}-\frac{4D}{3\Delta x^2}\right)C(i,n_x-1) &= R(i-1,n_x-1)+\frac{C(i-1,n_x-1)}{\Delta t}
\end{aligned} \tag{B4}$$

Equation (B4) can be expressed using a matrix and vectors.

$$\mathbf{Mc} = \mathbf{r} \tag{B5}$$

where

$$\mathbf{M}=\begin{bmatrix} m_{11} & m_{12} & 0 & 0 & \cdots & 0 & 0 \\ m_{21} & m_{22} & m_{23} & 0 & \cdots & 0 & 0 \\ m_{31} & m_{21} & m_{22} & m_{23} & 0 & \cdots & 0 \\ \vdots & \vdots & \vdots & \vdots & \ddots & & \vdots \\ \vdots & \vdots & \vdots & \vdots & & \ddots & \vdots \\ 0 & 0 & \cdots & m_{31} & m_{21} & m_{22} & m_{23} \\ 0 & 0 & \cdots & \cdots & m_{31} & m_{21}+\dfrac{D}{3\Delta x^2} & m_{22}-\dfrac{4D}{3\Delta x^2} \end{bmatrix}, \mathbf{c}=\begin{bmatrix} C(i,1) \\ C(i,2) \\ C(i,3) \\ \vdots \\ C(i,n_x-3) \\ C(i,n_x-2) \\ C(i,n_x-1) \end{bmatrix} \text{ and } \mathbf{r}=\begin{bmatrix} R(i-1,1)+\dfrac{C(i-1,1)}{\Delta t}+U\dfrac{3C(i,0)}{2\Delta x}+D\dfrac{C(i,0)}{\Delta x^2} \\ R(i-1,2)+\dfrac{C(i-1,2)}{\Delta t}-U\dfrac{C(i,0)}{2\Delta x} \\ R(i-1,3)+\dfrac{C(i-1,3)}{\Delta t} \\ \vdots \\ R(i-1,n_x-1)+\dfrac{C(i-1,n_x-1)}{\Delta t} \end{bmatrix}$$

where $m_{11}=\dfrac{1}{\Delta t}+\dfrac{4U}{2\Delta x}+\dfrac{2D}{\Delta x^2}$, $m_{12}=-\dfrac{U}{2\Delta x}-\dfrac{D}{\Delta x^2}$, $m_{21}=-\dfrac{4U}{2\Delta x}-\dfrac{D}{\Delta x^2}$, $m_{22}=\dfrac{1}{\Delta t}+\dfrac{3U}{2\Delta x}+\dfrac{2D}{\Delta x^2}$, $m_{23}=-\dfrac{D}{\Delta x^2}$, $m_{31}=\dfrac{U}{2\Delta x}$

$R(i-1,j)=f\left(C_{p1}(i-1,j),C_{p2}(i-1,j),C_{p3}(i-1,j),C_{p4}(i-1,j),C_{p5}(i-1,j)\right)$

Therefore the concentration vector **c** at time *i* can be obtained by:

$$\mathbf{c}=\mathbf{M}^{-1}\mathbf{r} \tag{B6}$$

This procedure is repeated until *i*=*nt*, as shown in Figure S1, and the flow velocity *U* is updated using the new permeability value which is dependent on the extent of lysis.

C. Grid Independence Test

A grid independence test has been carried out to ensure the accuracy of numerical solutions for the 1D transport and clot lysis model. The clot is of 5 mm in length with its front face located at the entrance of the occluded artery. For simplicity, the inlet concentration of tPA is set to 0.025μM and those of other fibrinolytic proteins are the same as their initial concentrations. Simulation results are compared in terms of lysis time and computation time. The result with the finest grid (No. 5 in Table S3) is considered as the exact solution, and all the tested grids result in negligible difference in lysis time, but there is an enormous difference in computation time. Therefore, the grid sizes shown in No. 1 are adopted throughout the work.

Table S3. Summary of grid test results.

	Grid sizes		Simulation results	Relative errors to the finest grid	Computation
	Δx [mm]	Δt [s]	Lysis completion time [min]	in lysis time [%]	time [s]
1	0.1	0.075	21.70	−0.50	46
2	0.05	0.075	21.76	−0.23	124
3	0.1	0.05	21.70	−0.50	75
4	0.05	0.05	21.76	−0.23	197
5	0.01	0.05	21.81	na	1,489

D. Comparison with Experimental Data in the Literature

Four clinical studies have been found where detailed PK data were reported. The dosage regimens used in the literature are simulated using our model and the results are compared with the reported patients' data. The kinetic parameters for the systemic PKPD model are also taken from the literature, which were derived from experimental data (Table S1). Only one parameter related to FBG degradation in plasma, $k_{3,cat}$, is adjusted as using the literature value would significantly underestimate the extent of FBG degradation, i.e., a value of 250 is adopted instead of 25 found in the literature (refer to Table S1).

D.1. Data from Tienfenbrunn et al. [S3]

Their data were acquired from patients with coronary thrombosis and peripheral arterial occlusion. Measurements were made for tPA and FBG only. Two sets of data were extracted: Group 1 with 25 mg of tPA infused over 7 hrs shown in Figure S2 and Group 2 with 60 mg of tPA infused over 90 min shown in Figure S3. The PLG level at the end of infusion for Group 1 is overpredicted by approximately 50%, while the predicted final PLG level for Group 2 is similar to the measured value. The FBG levels predicted by the model with the adjusted parameter for both groups are in reasonably good agreement with the measurements.

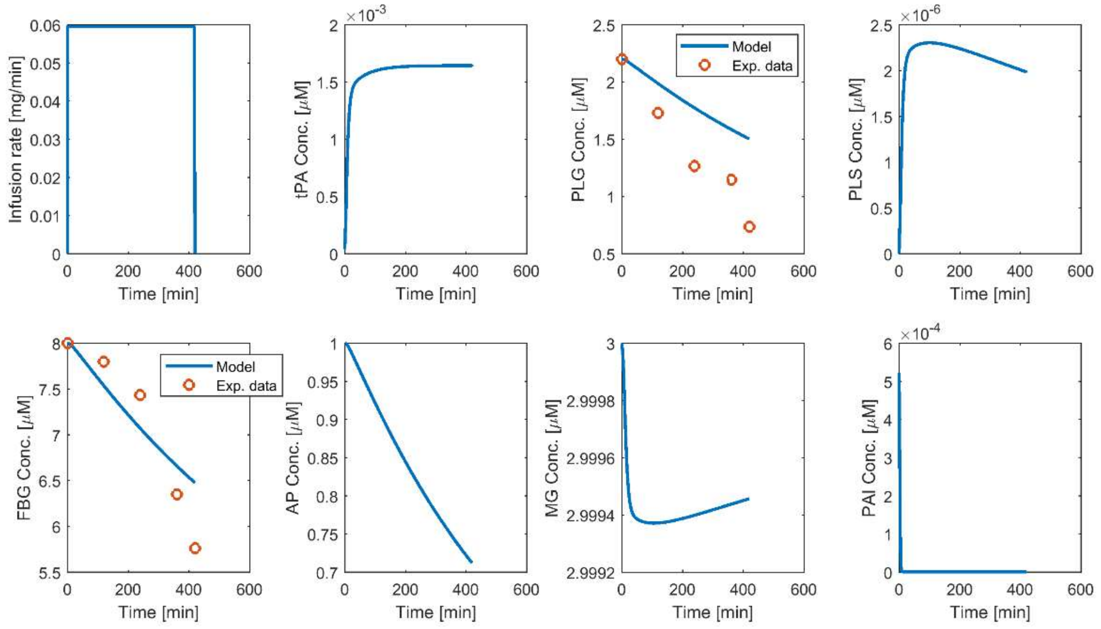

Figure S2. Data presented in Tienfenbrunn et al. [S3] with 25 mg of tPA infused over 7 h.

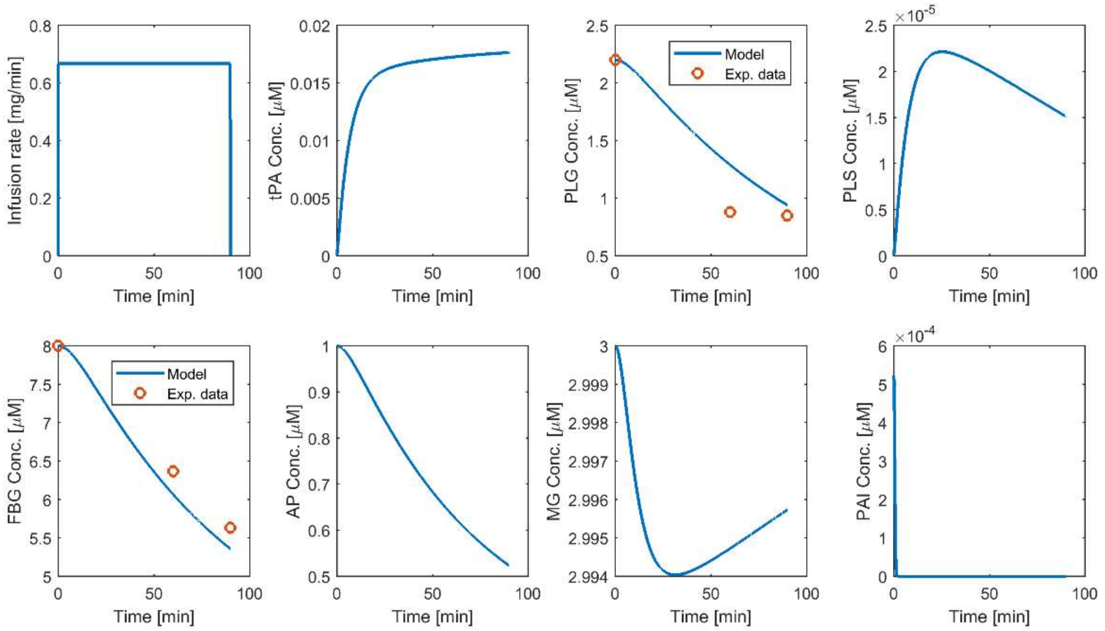

Figure S3. Data presented in Tienfenbrunn et al. [S3] with 60 mg of tPA infused over 90 min.

D.2. Data from Collen et al. [S8]

Collen et al. studied patients with acute myocardial infarction treated with 0.75 mg/kg of tPA over 90 min. Measured levels of PLG, FBG and AP *in vitro* are compared with model predictions, as shown in Figure S4. For all three proteins, the model predicts slightly higher final concentrations than the measured values. However, it was also pointed out by Collen et al. that there might have been further degradation of

these proteins *in vitro* in plasma samples due to remaining tPA, unlike *in vivo* in plasma. Therefore we have not further tuned the kinetic parameters for the reactions involving PLG and AP.

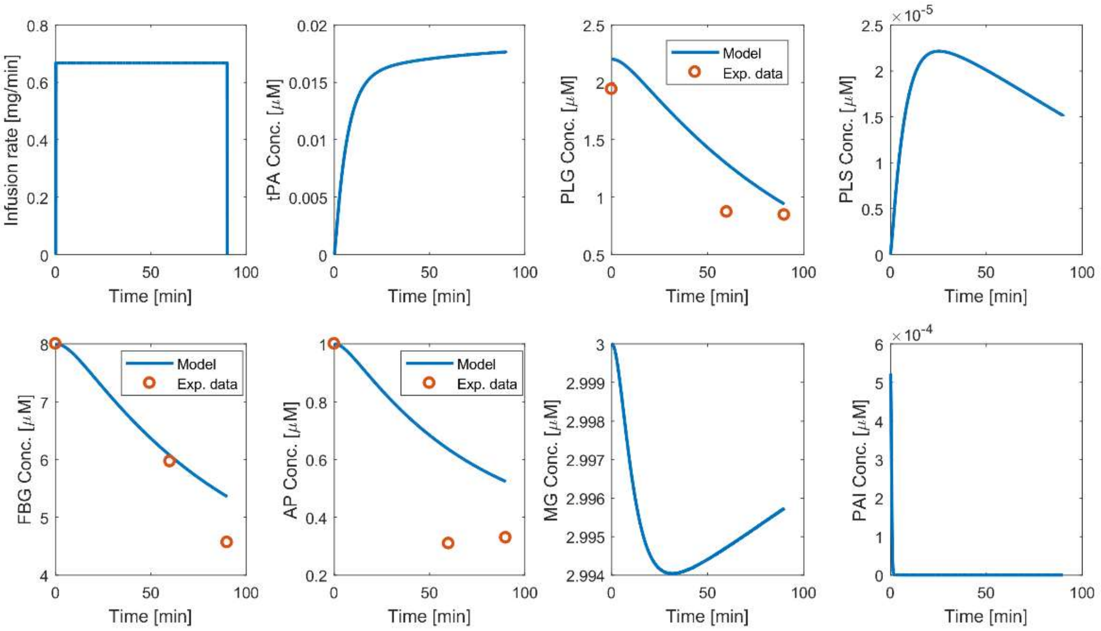

Figure S4. Data presented in Collen et al. [S8] with 0.75 mg/kg of tPA over 90 min.

D.3. Data from Tanswell et al. [S9]

Concentrations of tPA in healthy subjects were collected by Tanswell et al. during intravenous infusion of tPA. Two doses were studied: 0.25 mg/kg and 0.5 mg/kg over 30 min in Figures S5 and S6, respectively. Without tuning any parameters for tPA, the model predictions match the measured data very well.

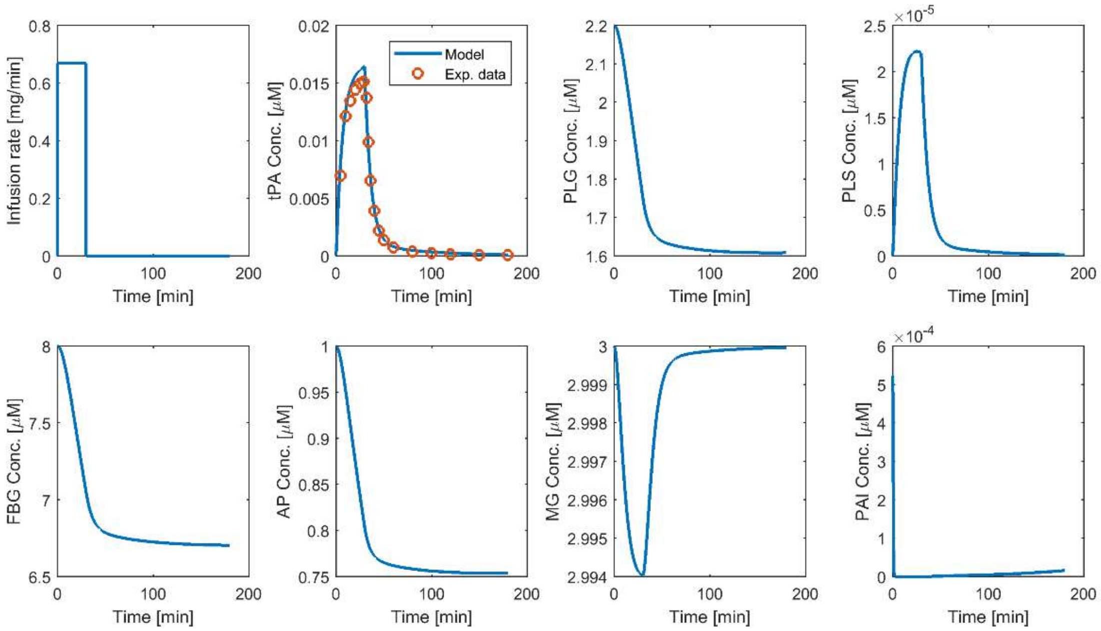

Figure S5. Data presented in Tanswell et al. [S9] with tPA dose of 0.25 mg/kg over 30 min.

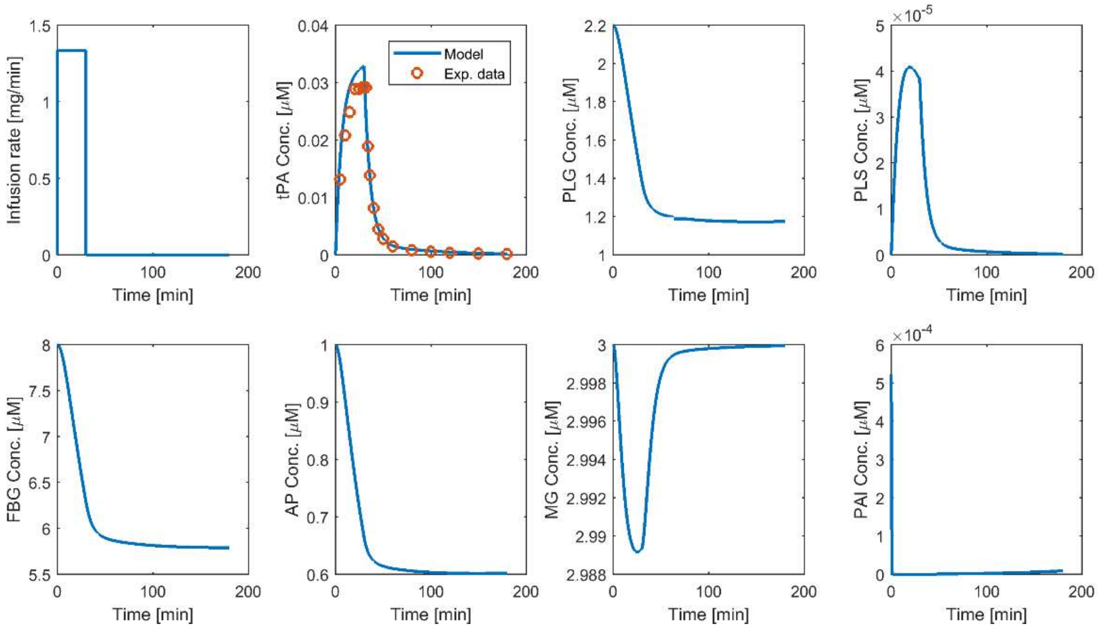

Figure S6. Data presented in Tanswell et al. [9] with tPA dose of 0.5 mg/kg over 30 min.

D.4. Data from Tebbe et al. [S7]

Patients with acute myocardial infarction were studied by Tebbe et al. to evaluate the efficacy of a single bolus thrombolysis. Predicted tPA and FBG concentrations are compared to the measured tPA and FBG levels. The level of tPA predicted by the model is in line with the measured values, while FBG exhibit significant discrepancy. Nonetheless, evidence to claim that the model and parameters are inappropriate is lacking, as measurements of FBG concentrations during thrombolysis are not available.

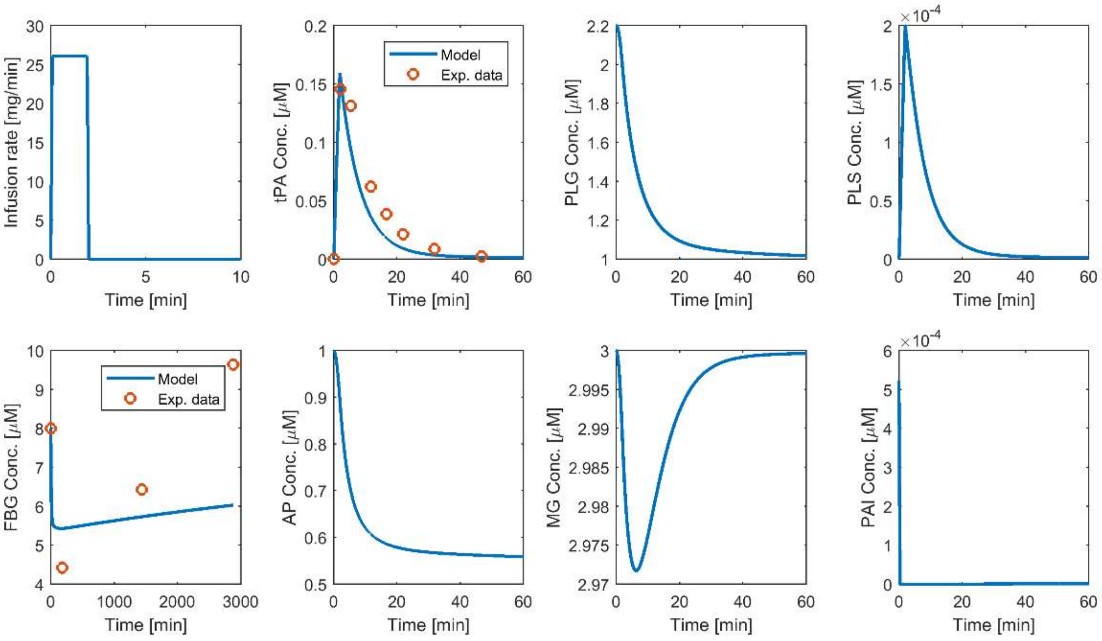

Figure S7. Data presented in Tebbe et al. [S7] with a single bolus of 50 mg of tPA over 2 min.

Reference

S1. Piebalgs, A.; Gu, B.; Roi, D.; Lobotesis, K.; Thom, S.; Xu, X.Y. Computational Simulations of Thrombolytic Therapy in Acute Ischaemic Stroke. *Sci. Rep.* **2018**, *8*, 15810.

S2. Sobel, B.E.; Gross, R.W.; Robinson, A.K. Thrombolysis, clot selectivity, and kinetics. *Circulation* **1984**, *70*, 160–164.

S3. Tiefenbrunn, A.J.; Graor, R.A.; Robison, A.K.; Lucas, F.V.; Hotchkiss, A.; Sobel, B.E. Pharmacodynamics of tissue-type plasminogen activator characterized by computer-assisted simulation. *Circulation* **1986**, *73*, 1291–1299.

S4. Chandler, W.L.; Alessi, M.C.; Aillaud, M.F.; Henderson, P.; Vague, P.; Juhan-Vague, I. Clearance of tissue plasminogen activator (TPA) and TPA/plasminogen activator inhibitor type 1 (PAI-1) complex: Relationship to elevated TPA antigen in patients with high PAI-1 activity levels. *Circulation* **1997**.

S5. Gorlatova, N.V.; Cale, J.M.; Elokdah, H.; Li, D.; Fan, K.; Warnock, M.; Crandall, D.L.; Lawrence, D.A. Mechanism of inactivation of plasminogen activator inhibitor-1 by a small molecule inhibitor. *J. Biol. Chem.* **2007**, *282*, 9288–9296.

S6. Jensen, P.E.H.; Humle Jørgensen, S.; Datta, P.; Sørensen, P.S. Significantly increased fractions of transformed to total α2-macroglobulin concentrations in plasma from patients with multiple sclerosis. *Biochim. Biophys. Acta—Mol. Basis Dis.* **2004**, *1690*, 203–207.

S7. Tebbe, U.; Tanswell, P.; Seifried, E.; Feuerer, W.; Scholz, K.H.; Herrmann, K.S. Single-bolus injection of recombinant tissue-type plasminogen activator in acute myocardial infarction. *Am J Cardiol* **1989**, *64*, 448–453.

S8. Collen, D.; Bounameaux, H.; De Cock, F.; Lijnen, H.R.; Verstraete, M. Analysis of coagulation and fibrinolysis during intravenous infusion of recombinant human tissue-type plasminogen activator in patients with acute myocardial infarction. *Circulation* **1986**, *73*, 511–517.

S9. Tanswell, P.; Seifried, E.; Su, P.C.A.F.; Feuerer, W.; Rijken, D.C. Pharmacokinetics and systemic effects of tissue-type plasminogen activator in normal subjects. *Clin. Pharmacol. Ther.* **1989**, *46*, 155–162.
